# Human Papillomavirus: A Narrative Review for Dental Providers in Prevention and Care

**DOI:** 10.3390/ijerph22030439

**Published:** 2025-03-17

**Authors:** Martin S. Lipsky, Geo Wolfe, Brisa A. Radilla, Man Hung

**Affiliations:** 1 Institute on Aging, Portland State University, Portland, OR 97201, USA; 2 College of Dental Medicine, Roseman University of Health Sciences, Summerlin, NV 89135, USA; 3 Portland State University, Portland, OR 97201, USA; 4 Division of Public Health, University of Utah, Salt Lake City, UT 84112, USA; 5 Department of Orthopaedics, University of Utah, Salt Lake City, UT 84112, USA; 6 Huntsman Cancer Institute, Salt Lake City, UT 84112, USA

**Keywords:** HPV prevention, oropharyngeal cancer, dental professionals, vaccination advocacy, early detection

## Abstract

(1) Introduction: Human papillomavirus (HPV) is a significant public health concern associated with various cancers, including a rising incidence of oropharyngeal cancer (OPC). Despite the availability of effective vaccines targeting high-risk HPV types, vaccination rates remain suboptimal. Dental professionals are uniquely positioned to contribute to HPV prevention through education, vaccination advocacy, and early detection. (2) Methods: This narrative review synthesized the literature from 2006 to 2024 on HPV epidemiology, pathophysiology, vaccine efficacy, and the role of dental providers in HPV prevention. Sources included peer-reviewed articles listed in Pubmed and Google Scholar, including observational studies and review articles, guidelines, clinical trials, and governmental data. Key barriers to HPV-related care in dental practice and strategies for overcoming them were analyzed. (3) Results: The review underscores the critical role of dentists in HPV-related disease prevention, highlighting their ability to detect HPV-related lesions, promote vaccine uptake, and address patient concerns. Barriers such as limited knowledge, discomfort discussing HPV, and misinformation were identified. Strategies like incorporating HPV education into dental training, utilizing emerging diagnostic tools, and adopting effective communication approaches can enhance the role of dental professionals in reducing HPV-associated cancer risks. (4) Conclusion: By integrating HPV education, vaccination advocacy, and early detection into routine dental care, dental professionals can play a transformative role in public health. A dental provider’s endorsement can increase HPV vaccine uptake and help prevent oral cancer. These efforts align with broader health promotion goals, offering a significant opportunity to reduce the burden of HPV-associated cancers and improve long-term patient outcomes.

## 1. Introduction

HPV is the most common sexually transmitted infections (STI) in the United States (U.S.) and worldwide [1,2]. Over 42.5 million Americans are infected with HPV [2], and each year, at least 13 million acquire new infections [3]. The prevalence of HPV in women without cervical abnormalities is 11–12%, with higher rates in sub-Saharan Africa (24%), Eastern Europe (21%), and Latin America (16%), while an estimated one in three men are infected with HPV and one in five are infected with a high risk, or oncogenic, HPV type. Although most infections spontaneously resolve [4,5,6], some individuals develop HPV-associated cancer, such as cervical, vulvar, vaginal, penile, or anal cancer. Globally, over 600,000 cancers, representing almost 5% of new cancer cases, are attributed to HPV [7,8]. In the United States alone, about 45,000 HPV-associated cancers are reported annually, and about 80% of those cancers are attributable to HPV [9].

The impact of HPV infection in females and its connection to cervical cancer is well-recognized. However, HPV is also strongly linked to oropharyngeal cancers (OPCs) in both men and women. Although HPV-related cervical and vaginal cancer rates have decreased since 1999 [7], the incidence of oral cancers has increased, and OPC is now the most common HPV-associated cancer [10]. Each year, there are more than 20,000 cases of OPC in the U.S. [11], and 70% of these are associated with HPV [12,13]. Although several HPV subtypes are considered high risk, HPV subtypes 16 and 18 account for most HPV-related OPC. Since the HPV vaccine protects against these viral subtypes, research suggests that HPV vaccination can help prevent and reduce the burden of OPC [14]. Unfortunately, HPV vaccine coverage often falls short of target levels [15], suggesting opportunities for the primary prevention of OPC may be missed.

Dental providers provide secondary screening for the signs and symptoms of OC as a routine component of dental care. However, despite recommendations by the American Dental Association (ADA) and the American Academy of Pediatric Dentistry (AAPD) to promote HPV vaccination, few dentists incorporate primary OPC prevention by advocating for HPV vaccination [16]. Dentists and oral hygienists report a lack of knowledge about HPV and the HPV vaccine as one barrier to incorporate vaccine education and advocacy into their clinical practice [16,17]. This narrative review seeks to address this knowledge gap by presenting a succinct overview of HPV and the HPV vaccine relevant for oral health providers, documenting its link to oral health, and how dentists may aid in HPV prevention. Understanding the link between HPV and OPC can enhance a dental professional’s confidence in recommending the HPV vaccine and counseling about other HPV prevention measures. Endorsement increases vaccine uptake [18], and dental professionals have an opportunity to play a principal role in advocating for HPV vaccination and oral cancer prevention.

## 2. Methods

A narrative literature review employs a non-systematic review strategy to provide an overview and up-to-date knowledge about a specific topic [19]. Narrative reviews are useful for broad topics, in contrast to systematic reviews, which are better suited to a specific question or narrower focus [20,21]. For healthcare professionals, a narrative review can efficiently condense large amounts of information from a wide range of sources about a patient care topic issue into just a few pages [22] and is suitable for summarizing the literature for a complex topic such as HPV [23].

To organize and synthesize articles related to the narrative review of HPV information relevant to oral health providers, two authors (MSL and MH) independently reviewed the HPV literature using the MESH terms human papillomavirus, oral health, oral medicine, and cancer vaccines to develop an initial review outline. The two authors shared their proposed outlines and, after discussion, developed a draft outline to share with the study team. The study team reviewed the outline, and their suggestions were reviewed by the senior authors (MSL and MH) and discussed until they achieved a consensus on the final outline for the review.

The authors then conducted PubMed and Google Scholar searches for English-language articles published between 1 January 2006 and 31 December 2024 about HPV epidemiology, pathophysiology, HPV vaccine, and relevance to oral health. The authors chose 2006 because the HPV vaccine was first licensed in the U.S.

The authors reviewed the retrieved articles for relevance and the bibliographies of the identified manuscripts for articles and other data sources potentially relevant to the review outline. The authors (MSL, GW, BAR, and MH) considered all types of peer-reviewed and full-length studies in English, prioritizing recent guidelines, clinical trials, other original research, and comprehensive, high-quality reviews for inclusion. When appropriate, governmental websites such as the Centers for Disease Control and Prevention, the National Institutes of Health, and other high-quality websites were used to augment the search and as statistical and epidemiologic data sources. The 88 HPV-related sources for this review included 6 guidelines, 1 consensus document, 39 original research papers, 29 review articles, 6 government-sponsored websites, and 7 other sources, including 1 case study, 1 pharmaceutical website, 3 university- and hospital-sponsored websites, and 2 websites sponsored by national organizations.

To guide the development of the manuscript, the authors used a critical appraisal tool called SANRA (Scale for the Assessment of Narrative Review Articles) to enhance their internal review process and improve the quality of the final manuscript. SANRA recommends a quality checklist for narrative reviews that includes the following: (a) an explanation of the review’s importance/relevance, (b) a statement of the aims of the review, (c) a description of the literature search, (d) targeted referencing, (e) solid logic or scientific reasoning, (f) adequate presentation of relevant, and (g) appropriate endpoint data and conclusions [24]. Two authors (MH and MSL) assessed the final manuscript to confirm it met the SANRA criteria.

## 3. Results and Discussion

### 3.1. Human Papillomavirus

Human papillomavirus (HPV) is a double-stranded DNA virus with more than 225 identified types classified into five genera: alpha, beta, gamma, mu, and nu. HPV primarily infects the basal epithelium of the skin and mucosal tissues. Approximately 40 types are transmitted through direct sexual contact, affecting the genital areas, mouth, and throat, with ongoing efforts to organize HPV research through standardized genotyping and reference resources [25].

Virologists usually divide HPV into two groups: low-risk types, which can cause skin and genital warts, and high-risk types, like HPV 16 and 18, which are strongly linked to cancers of the cervix, throat, and genital areas. Table 1 summarizes the HPV connection by cancer site.

In the oral cavity, low-risk types of HPV infections may be asymptomatic or can cause verruca vulgaris (common warts), squamous papillomas, condyloma acuminatum, and rarely benign oral hyperplasia [26]. In contrast, high-risk HPV types are linked to cancer, with HPV subtypes 16 and 18 alone accounting for 80 to 90% of HPV-positive oropharyngeal cancers [27]. Table 2 summarizes the percentages of HPV-Attributable OPC by subtype. Understanding how HPV spreads and its natural history is important for public health efforts to control it.

HPV infection develops when viral particles infect the basal epithelium through micro-abrasions or epithelial wounding of the mucosal lining of the mouth, throat, respiratory, or anogenital tract [28]. HPV spreads through direct contact, most commonly by sexual contact, and oral sex is the main transmission mode of oral HPV infection, and the risk increases with the number of oral sexual partners [29,30,31]. Using condoms and dental dams during oral sex may help reduce the risk of infection [32,33,34], HPV can also spread from an infected mother to her child during childbirth or from one part of your body to another (autoinoculation) [35]. While possible, the lack of concordance of oral and cervical HPV infections suggests autoinoculation is an uncommon means of acquiring oral HPV [35].

Most individuals become infected with HPV soon after becoming sexually active, and 75 to 80% of sexually active people contract HPV during their lifetime [36]. In oral infections, few develop symptoms, and most individuals clear the virus within 1 to 2 years [37,38]. However, some individuals develop a persistent infection that may last for years. Risk factors for failing to clear HPV include smoking and HIV, suggesting that tobacco-related and HIV-related immunosuppression may impact the natural history of oral HPV [39]. Other risk factors for oral HPV include the number of lifetime sex partners, sex at a younger age, poor oral hygiene and smoking. Men and older individuals are also at greater risk for oral HPV persistence (see Table 3) [40,41,42,43]. Persistent high-risk HPV infection triggers deregulated viral gene expression, leading to excessive cell proliferation, deficient DNA repair, and the accumulation of genetic damage, which promotes malignant transformation in infected cells [44].

No cure exists for the virus, which underscores the importance of prevention. Treatment is directed at managing symptoms and depends on the area involved and the extent of the lesions. Modalities include surgical excision, laser ablation, cryosurgery, electrosurgery, and immunostimulants (e.g., interferon). For recalcitrant lesions, topical cidofovir may be effective.

### 3.2. Importance to Oral Care

HPV-related oral lesions are typically small, benign, wart-like growths called squamous papillomas that appear on the surfaces of the mouth or oropharynx [45]. These lesions can be found on the tongue, palate, throat, or other oral tissues and are associated with low-risk HPV types—especially HPV-6 and HPV-11. However, infections with high-risk HPV types, particularly HPV-16 and HPV-18, require closer monitoring, as these strains can induce cellular changes that may ultimately lead to cancer [46].

High-risk HPV strains exert their carcinogenic effects through the expression of E6 and E7 early proteins, which interfere with key tumor suppressors in host cells [47]. E6 primarily targets the p53 tumor suppressor, inhibiting its role in DNA repair and apoptosis. Meanwhile, E7 binds to the retinoblastoma protein (pRB), disrupting its control over the cell cycle. Only high-risk HPV E6 and E7 can bind to p53 or pRb, confirming that HPV-related cancers originate from high-risk HPV types [48]. Interference with the tumor suppressor pathways leads to uncontrolled cell growth, genomic instability, and the accumulation of mutations that drive cancer progression [49]. Additionally, E6 activates telomerase, an enzyme that maintains chromosomal integrity, further promoting cellular immortalization. The combined actions of E6 and E7 deregulate pathways involved in immune response, epithelial differentiation, and survival signaling, which promote HPV-related oncogenesis [50].

Over the past two decades, HPV has emerged as a significant risk factor for oropharyngeal squamous cell carcinoma (OPSCC), with the incidence of HPV-positive (HPV+) tumors in high-income countries increasing from under 20% to more than 70% of all oropharyngeal cancers [51,52]. In some high-income countries, HPV-associated oral cancer exceeds cervical cancer as the leading HPV-associated cancer. This uptick is especially notable among younger and non-smoking individuals, underscoring the need for heightened awareness within the dental community. Since HPV+ OPSCC often presents with subtle or no early symptoms, it is commonly diagnosed at advanced stages when treatment can be more complex with less favorable outcomes [53]. HPV+ OPSCC is less strongly linked to alcohol and tobacco than HBV- OPSCC [54,55]. Table 4 summarizes the differences between HPV+ and HPV- oral cancers.

A thorough visual and tactile exam relying on clinician expertise remains the first line of early detection [56]. Advancements in diagnostic technology offer the promise of more effective tools for identifying HPV-related lesions, especially those in the oropharynx. For example, augmenting clinical exams with artificial intelligence (AI) and photographic images may be a more accurate and quicker method for diagnosing oral cancer in its early stages [57,58]. AI can analyze imaging data, detect subtle patterns that may not be immediately apparent to the human eye, and improve diagnostic precision. Machine learning algorithms trained on large datasets of oral lesions have promise in distinguishing benign from malignant changes with high sensitivity and specificity. Future research will likely refine these models, incorporating multi-modal data such as imaging, clinical history, and molecular markers to enhance predictive accuracy and clinical applicability.

Salivary diagnostics potentially offer a non-invasive and increasingly validated method of detecting high-risk HPV strains [59]. However, while testing demonstrates an active HPV infection, some experts question its utility in screening for HPV-related OPC since most individuals either clear the infection or fail to progress to malignancy [60]. Tools such as fluorescence visualization devices might improve diagnostic accuracy by helping clinicians identify lesions in areas of the throat that are difficult to inspect [61]. Currently, most national organizations, including the US Preventive Services Task Force, do not recommend population-based screening due to insufficient evidence that screening reduces oral cancer mortality [62,63]. However, screening high-risk groups with one of the emerging techniques may be cost-effective and provide opportunities for earlier diagnosis and improved outcomes for individuals vulnerable to HPV-related oropharyngeal carcinoma. Further research is needed to develop guidelines for using these new tools.

### 3.3. HPV Vaccine

The HPV vaccine—particularly the 9-valent HPV vaccine Gardasil-9—represents a critical tool in the prevention of HPV-related cancers. The vaccine protects against nine HPV types, including those responsible for the majority of cervical, oropharyngeal, and other anogenital cancers [64]. Administered as a two- or three-dose series, depending on the age of initiation, the vaccine is most effective when given before HPV exposure, typically recommended at ages 11–12 but can be given as early as nine years old [65]. Routine vaccination is recommended up to age 26, but unvaccinated individuals from age 27 to 45 at risk of a new HPV infection may benefit from vaccination. In contrast, those in long-term, monogamous relationships are at low risk for a new infection and are less likely to benefit [66]. The following outlines the CDC-recommended HPV 9 -valent recombinant vaccine (Gardasil-9) schedule and precautions (https://www.cdc.gov/vaccines/vpd/hpv/hcp/recommendations.html, accessed on 3 March 2025).

- Administer intramuscularly.
- Vaccination initiation is recommended at age 11 or 12 years (can begin at age 9).
- Two doses of HPV vaccine are recommended for most persons starting the series before their 15th birthday.
  ◦ The second dose of HPV vaccine should be given 6 to 12 months after the first dose.
  ◦ Adolescents who receive two doses less than 5 months apart require a third dose of HPV vaccine.
- Three doses of HPV vaccine are recommended for ages 15 through 45 years, and for immunocompromised persons.
  ◦ The recommended three-dose schedule is 0, 1–2, and 6 months.
  ◦ Three doses are recommended for immunocompromised persons (including those with HIV infection) aged 9 through 26 years.
- Minor illnesses should not delay vaccination.
- Vaccination should be delayed in moderately or severely ill individuals until symptoms improve.
- Pregnant women should defer vaccination until they are no longer pregnant.

Clinical trials and population-level studies have established the high efficacy of HPV vaccines [67,68]. More than 98% of recipients develop an antibody response to targeted HPV types after completing the vaccination series. Studies also demonstrate good immunogenicity after a single shot, and in a 2022 position paper, the World Health Organization (WHO) stated that a single-dose (off-label) schedule provides similar efficacy and durability of protection as a two-dose schedule [69,70]. Consequently, WHO recommends that an off-label single-dose schedule can be used in girls and boys aged 9–20 years and two doses with a 6-month interval after age 21. Immunocompromised individuals should receive a minimum of two doses and, when possible, three doses [71].

Since the vaccine’s introduction in 2006, infections with HPV types associated with cancers have decreased significantly—with an 88% reduction among adolescent girls and an 81% reduction among young women [72]. Other studies confirm that vaccination reduces oral HPV infection [73,74,75], and in June 2020, the U.S. Food and Drug Administration expanded the indication for the HPV 9-valent vaccine to include the prevention of OPC head and neck cancers caused by HPV [76]. However, because of the long lag between infection and cancer and incomplete HPV vaccination uptake, significant reductions in the OC may not be seen until after 2045 [77].

Safety profiles for the HPV vaccine are highly favorable, with extensive monitoring and post-licensure evaluations affirming its safety across diverse populations [64]. The vaccine uses noninfective virus-like particles, and since it does not contain live virus or viral DNA, the vaccine cannot cause HPV infection or oncogenesis. Side effects are generally mild and transient, commonly including mild reactions such as pain, redness, or swelling at the injection site, as well as occasional fever or headache. Severe adverse events, including anaphylaxis, are rare. A minor acute illness (e.g., diarrhea or mild upper respiratory tract infection, with or without fever) is not a reason to defer vaccination. However, vaccine administration should be delayed in a moderately or severely ill person until symptoms improve. Vaccination should be withheld in pregnant women until after they are no longer pregnant [78,79].

Despite its demonstrated safety and efficacy, only 58.6% of adolescents aged 13–15 years received 2 or 3 doses of human papillomavirus (HPV) vaccine in 2022 [80], which falls short of the Healthy People 2030 goal of 80% [81]. Unfortunately, HPV vaccine uptake has not improved since the pandemic [82]. Common themes contributing to vaccine hesitancy include misinformation about HPV vaccines, including unfounded concerns about adverse health effects, mandatory vaccinations, and vaccine efficacy [83]. Social media platforms amplify these concerns by giving misinformation higher visibility than accurate content. For instance, Kornides et al. [83] observed that nearly one-quarter of #HPV tweets contained misleading claims, and these posts received more than five times the number of retweets compared to supportive messages—demonstrating the substantial reach and influence of misinformation in fueling vaccine hesitancy. Oral health providers should be prepared to address common myths surrounding the HPV vaccine, such as safety concerns, that PAP smear testing obviates its value, that it encourages promiscuity and risky sexual behavior, and that boys do not need vaccination [84]. Oral health professionals, including dentists and dental hygienists, are uniquely positioned to advocate for HPV vaccination due to their frequent interactions with preteen and adolescent patients. Research indicates that many people not vaccinated for HPV visited a dental provider within the past year, underscoring the unique opportunity for providers to address vaccine-related questions and concerns [85].

Nevertheless, common barriers in dental settings include limited knowledge about HPV, communication challenges surrounding sexually transmitted infections, and uncertainty about whether discussing HPV vaccination falls within providers’ scope of practice [85,86]. To address these barriers, studies emphasize the importance of professional organization guidance, incorporating HPV content into training programs, and developing practical communication tools. Such strategies can empower oral healthcare professionals to discuss HPV prevention confidently and ultimately contribute to reducing HPV-related oropharyngeal and other cancers.

### 3.4. Patient Communication Tips

To effectively address HPV and its associated risks, healthcare professionals need communication strategies that promote understanding and reduce stigma [87]. Effective communication is crucial when discussing HPV vaccination, as its link to sexual health can make it uncomfortable for patients and oral health providers [16,88,89]. Research indicates that adopting a clear, empathetic and non-judgmental communication style when talking about HPV helps reduce patient anxiety, foster trust, and makes patients feel heard, respected, and empowered to make informed decisions about their health [90]. Unfortunately, discussions about HPV in the oral healthcare setting are infrequent [85] and some younger individuals may be uncomfortable with HPV-related discussions in a dental care setting [89].

Murciano-Gamborino et al. highlighted the importance of tailoring communication strategies to the needs of different population groups [90]. Healthcare professionals must recognize that parents, adolescents, and older adults, may approach HPV with different levels of understanding and concerns. For example, parents may worry about the safety or necessity of vaccinating their children, while adolescents may be more focused on cancer prevention rather than the sexual transmission of the virus [87,91]. By addressing these concerns specifically and empathetically, an oral healthcare professional can provide contextually relevant information about the vaccine’s role in preventing HPV-related cancers.

Pilkington et al. [92] provided valuable insights into the importance of communication when delivering difficult news, a skill that applies to discussions about HPV. One key element is ensuring that the conversation occurs in a supportive and respectful environment where patients feel safe expressing their concerns. When discussing the vaccine, healthcare professionals should acknowledge a patient’s questions or concerns, providing a space for open dialog. This approach helps patients feel more comfortable asking questions, such as those regarding the vaccine’s safety or its relevance to their health.

Pilkington et al. also emphasized the importance of using clear, accessible language when discussing sensitive health topics [92]. This principle is crucial when talking about HPV, as patients may not be familiar with medical terminology. Simplifying complex medical terms and using everyday language ensures that patients fully understand the risks associated with HPV and the benefits of vaccination. For instance, instead of using technical terms like “high-risk strains”, a healthcare provider might explain, “There are some types of HPV that can lead to mouth cancer, but the vaccine helps prevent this and other serious health problems”.

In conclusion, effective communication about HPV involves creating an open, empathetic dialog that respects patients’ concerns while providing them with the information they need to make informed choices about their health [88]. By employing clear, non-judgmental language and addressing specific concerns, healthcare professionals can reduce stigma and increase understanding, ultimately improving vaccine uptake and promoting better health outcomes.

### 3.5. Role of the Dentist

Dentists play a pivotal role in the prevention and early detection of HPV+ OPSCC and in promoting public awareness about HPV [93]. As primary healthcare providers who routinely examine the oral cavity, dentists are uniquely positioned to identify early signs of HPV-related lesions and suspicious oral cavity changes that may indicate malignancy. Their expertise in oral health allows them to detect subtle abnormalities during routine check-ups, facilitating timely referrals for medical evaluation and early intervention. Beyond detection, dentists serve as educators, bridging gaps in public knowledge by discussing the risks of HPV, the connection between HPV and oropharyngeal cancers, HPV in men, and the benefits of vaccination with patients and their families [94]. This educational role is especially crucial given the sensitive nature of HPV discussions, as dentists can provide accurate, evidence-based information in a nonjudgmental manner, addressing misconceptions and alleviating vaccine hesitancy.

Moreover, dentists are uniquely situated to advocate for HPV vaccination during regular dental visits [16], particularly for adolescents and young adults who may otherwise miss opportunities to receive information from other healthcare providers. They can enlist parents by emphasizing the safety and efficacy of the vaccine and framing it as a cancer-prevention measure. In addition to direct patient interactions, dentists can play a broader role in interdisciplinary collaboration, partnering with physicians, oncologists, and public health professionals to create a cohesive approach to HPV-related disease prevention [93,94]. Dentists can also engage in community outreach initiatives, participate in public health campaigns, and advocate for integrating HPV education into school-based oral health programs.

Incorporating HPV education, vaccination advocacy, and OC screenings into routine care not only enhances patient outcomes but also aligns with expanding the role of oral health providers in overall health promotion. By staying current on HPV research, adopting innovative diagnostic technologies in machine learning, and fostering open communication, dentists can actively contribute to reducing the burden of HPV-associated diseases, ultimately supporting the overarching goals of public health to improve population-wide cancer prevention.

## 4. Limitations

This review has limitations. In a non-systematic review there can be selection bias in article inclusion and that key studies may be overlooked. Also, the quality of the included evidence was not systematically assessed. Despite these limitations it is important to recognize that narrative reviews are a useful tool for summarizing and synthesizing a large body of information on a broad topic.

## 5. Conclusions

Dentists can play a crucial role in the fight against HPV+ OPSCC by leveraging their unique position as oral healthcare providers to promote prevention, early detection, and education. With the rising incidence of HPV+ OPSCC, the dental community must embrace opportunities to discuss HPV with patients, advocate for vaccination, and recognize early signs of oral and oropharyngeal lesions. Incorporating HPV-related education and screening into routine dental care can not only improve patient outcomes but also contribute to broader public health initiatives aimed at reducing the prevalence of HPV-related cancers. By staying informed and proactive, dentists can help bridge the gap between oral health and systemic disease prevention, ultimately playing a vital role in improving long-term health outcomes in their communities.

## Figures and Tables

**Table 1:** HPV-associated cancers by tumor site.

Site	% Linked to HPV
Anal	90
Cervical	90
Vaginal	75
Vulvar	70
Penile	60

Source: National Cancer Institute https://www.cancer.gov/about-cancer/causes-prevention/risk/infectious-agents/hpv-and-cancer#what-cancers-are-caused-by-hpv-infection (accessed on 22 February 2025).

**Table 2:** Estimated annual HPV-attributable oropharyngeal cancer cases by subtype.

Average Annual Cases (2012–2016)	Number (%) Probably Caused by Any HPV Type	Number (%) Caused by HPV Subtypes 16 and 18	Number (%) Caused by HPV Subtypes 31/33/45/52/58
Total 16,479	11,600 (70%)	9900 (60%)	900 (6%)
Female 3203	2000 (63%)	1600 (51%)	300 (10%)
Male 13,276	9600 (72%)	8400 (63%)	300 (4%)

Adapted from [9].

**Table 3:** Risk factors for oral HPV.

Risk Factor	Description
Smoking	Linked to increased susceptibility to HPV infection and potential persistence.
Oral sex	The primary mode of HPV transmission to the oral cavity.
Number of sexual partners	A higher number of lifetime partners increases exposure risk.
Younger age at first encounter	Early sexual debut is associated with a greater likelihood of acquiring HPV.
HIV infection	Immunosuppression increases the risk of persistent HPV infection.
Older age	HPV infections are more likely to persist with age due to immune system changes.
Sexual behavior	Oral HPV incidence is higher in individuals with more oral sex partners, and transmission is observed in both heterosexual and MSM populations.

Derived from: [41].

**Table 4:** Differences between HPV-positive and HPV-negative oropharyngeal cancers.

Characteristic	HPV-Positive OPSCC	HPV-Negative OPSCC
Sexual risk factors	Oral Sex, increased number of partners	Not related
Incidence	Increasing	Decreasing
Prognosis	More favorable	Less favorable
Location	Tonsil, base of tongue	All sites
Common Presentation	Painless neck mass	Sore throat, dysphagia, and/or odynophagia
Age	Younger cohorts	Older cohorts
Smoking	Less strongly associated	More strongly associated
Alcohol	Less strongly associated	More strongly associated

## Data Availability

Not applicable.
